# Exploring the Association Between Intravenous Lorazepam and Mortality Among Older Hospitalized Patients With and Without Cognitive Impairment

**DOI:** 10.7759/cureus.72121

**Published:** 2024-10-22

**Authors:** Joseph H Flaherty, Riddhi R Patel, Anupama S Gangavati, Michael B Cannell

**Affiliations:** 1 Geriatrics, Envision Physician Services, Dallas, USA; 2 Internal Medicine/Geriatrics, University of Texas Southwestern Medical School, Dallas, USA; 3 Epidemiology, Human Genetics and Environmental Sciences, UTHealth Houston School of Public Health, Dallas, USA; 4 Public Health, University of Texas, Houston, USA; 5 Internal Medicine, Geriatrics Division, UT Southwestern Medical School, Dallas, USA

**Keywords:** adverse drug events, benzodiazepines, cognitive impairment, delirium, dementia, geriatrics, hospitalized older patients, iv lorazepam, mortality

## Abstract

Background

In a previously published study about the effects of an inpatient geriatrics program on mortality among older patients with and without cognitive impairment, intravenous (IV) lorazepam was unexpectedly found to be one of the variables associated with mortality in the multivariate analysis. The purpose of this study was to further explore the association between IV lorazepam and mortality.

Materials and Methods

This was a secondary data analysis of a previously published retrospective study. The setting was a 500-bed community-based hospital, Level-1 Trauma Center, and Stroke Center (Dallas, Texas, United States). Participants were all patients aged 70+ admitted between January 1, 2017, and December 31, 2019. Logistic regression was used to evaluate the association between IV lorazepam (defined as receiving ≥1 dose) and mortality (death during hospitalization) among patients with cognitive impairment [defined as in the original study using a list of >30 IInternational Classification of Diseases, Tenth Edition (ICD-10)] and without cognitive impairment. Covariables included age, gender, case mix index, ICU stay, sepsis, palliative care, oral benzodiazepines, oral and IV antipsychotics, and oral and IV opioids. Logistic regression was used to calculate the adjusted odds ratio (aORs) and 95% confidence intervals (CI) of mortality.

Results

Of 20,541 patients, 6,197 (30.2%) had cognitive impairment of which 1430 (23.1%) received IV lorazepam, with a mortality rate of 16.9%. Of 14,344 patients without cognitive impairment, 1,468 (10.2%) received IV lorazepam, with a mortality rate of 32.0%. After controlling for covariables, aORs for mortality among those who received IV lorazepam was 3.37 (95% CI: 2.52-4.50) for patients with cognitive impairment and 7.72 (95% confidence interval (CI): 6.09-9.79) without cognitive impairment. Even when ICU and palliative care patients were excluded, aOR for mortality remained high for those with (4.09; 95% CI: 2.17-7.69) and without cognitive impairment, 18.82 (95% CI: 13.39-26.46).

Conclusion

Despite the limitations of this exploratory study, including a lack of data on the dosage and duration of IV lorazepam, further research is warranted to examine the possible association between IV lorazepam and increased mortality among older hospitalized patients, both with and without cognitive impairment.

## Introduction

One of the most prescribed benzodiazepines (BDZs) for older hospitalized adults is lorazepam [1,2], often in intravenous (IV) form [3]. Inpatient use of BDZs is associated with an increased risk of falls, fractures, other injuries, and delirium [4-6]. However, the association between IV lorazepam and mortality among older hospitalized patients is not as clear [7-9]. Most studies have examined mortality risks among community-dwelling older adults [10,11], patients in intensive care units (ICU) [12], or patients receiving palliative care or hospice [13,14]. Some have found an increase in mortality risk [9,11,12,14] while others have not [7,8,10,13]. Furthermore, most studies investigated oral BDZ use [10,11] or were unclear if IV BDZs were used [7,8,14]. We found one study that investigated the use of IV lorazepam, but the mean age of the participants was 62 [13]. Lastly, none of these studies investigated potential differences between older patients with and without cognitive impairment, a condition potentially related to the use of BDZs and mortality risk.

In a previously published study about the effects of an inpatient geriatrics program on mortality among older patients with and without cognitive impairment, we found that IV lorazepam was more strongly associated with mortality than almost any other measured characteristic, as high as those receiving palliative care, and higher than those with sepsis or an ICU stay [3]. No other drug category in the study (oral BDZs, IV antipsychotics, oral antipsychotics, IV opioids, oral opioids) had the same magnitude of associated mortality. The primary aim of the present study is to further explore the previously observed relationship between IV lorazepam and mortality and to investigate differences in that relationship by cognitive status. 

This research was previously presented as a meeting abstract at the 2023 American Geriatrics Society Annual Scientific Meeting on May 6, 2023.

## Materials and methods

Data was obtained from the electronic medical record database (retrospective chart review) of a single hospital (500-bed community-based Level 1 Trauma Center and Stroke Center). The database included all 20,541 patients age 70+ admitted from January 1, 2017, through December 31, 2019 [3].

Variables

The independent variable of interest was IV lorazepam, which was defined as the use of the drug one or more times during hospitalization. It was not possible to capture individual dosages, the total amount used, or the reason for use.

The dependent variable was mortality which was defined as death during hospitalization.

Cognitive impairment was defined as the presence of at least one of 36 International Classification of Diseases, Tenth Edition (ICD-10) diagnosis codes that were consistent with any type of cognitive impairment (Appendix 1). Reasons for using the broader term cognitive impairment (instead of delirium, dementia, and delirium superimposed on dementia) and reasons for comparing patients with and without cognitive impairment are described elsewhere [3] and in Appendix 2.

Covariables included age, gender, case mix index (CMI), ICU stay, sepsis, palliative care, oral BDZ use, IV antipsychotic use, oral antipsychotic use, IV narcotic use, oral narcotic use, Foley catheter use, and an alcohol-related diagnosis. The rationale for the choice of variables is described elsewhere [3]. CMI measurements are calculated in various ways but typically utilize the relative DRG weight of a hospital's inpatient discharges. CMI measurements usually reflect the complexity and severity of a patient’s illnesses. For this study, it was chosen as a proxy for severity of illness. 

Data were complete except for the female/male sex [missing 49 (0.8%) among those with cognitive impairment and 165 (1.2%) among those without cognitive impairment].

Statistical methods

Descriptive means and percentages were calculated for all variables of interest. A two-sample t-test was used to test for differences in the mean of continuous variables and a chi-square test was used to test for differences in the frequency distributions of the categorical variables.

The unadjusted association between IV lorazepam use and mortality was estimated by fitting a logistic regression model where the primary exposure of interest was IV lorazepam use and the primary outcome of interest was mortality. The adjusted association between IV lorazepam use and mortality was calculated by including additional variables in the logistic regression model that were selected to statistically control for other factors that may influence the magnitude of the unadjusted estimate of association. Covariables were selected by first fitting an adjusted model that included all significant variables from the univariate analysis (i.e., the full model), then fitting a series of reduced models -- each omitting one of the candidate variables -- and then evaluating the independent contribution of the omitted variable to the full model using a likelihood ratio test.

Since the risk of mortality is relatively higher among patients receiving ICU care or palliative care services, and the use of IV lorazepam is potentially common among these groups of patients, all analyses were performed again excluding patients who received ICU care or palliative care services. All the analyses were completed using Stata 17.0 (StataCorp LLC, College Station, TX).

## Results

Out of 20,541 consecutively admitted patients age 70+ during the three-year period (2017-2019), 6197 (30.2%) had cognitive impairment, of which 1430/6197 (23.1%) received IV lorazepam (Table 1). The group who received IV lorazepam was slightly younger and had a lower percentage of women. The group also had a higher CMI, more often had an ICU stay, sepsis, and used palliative care services. The percentages of patients who received oral BDZs, IV antipsychotics, oral antipsychotics, and IV opioids were higher in this group. Foley catheter use and an alcohol-related diagnosis were also higher in the IV lorazepam group. Lastly, the mortality rate in the IV lorazepam group was higher [N=242; (16.9%)] compared to those who did not receive IV lorazepam [N=152; (3.2%)] (P<0.001) (Table 1), a roughly five-fold difference. 

**Table 1:** Descriptive characteristics of patients with cognitive impairment admitted to a community-based hospital, 2017 through 2019 (n = 6,197). The data in columns 2 and 3 have been represented as N (%) except for age and CMI, for which the data have been represented as Mean±SD. A two-sample t-test was used to test for differences in the mean of continuous variables and a chi-square test was used to test for differences in the frequency distributions of the categorical variables (column 5). P-values are shown in column 4 and considered significant if p<.05. Abbreviations: IV: intravenous; SD: standard deviation; CMI: case mix index; ICU: intensive care unit.

Characteristics	No IV Lorazepam (n = 4,767)	IV Lorazepam (n = 1,430)	P-value	T-test and chi-test values
	n (%)	n (%)		
Age (y) (Mean ± SD)	82.8 ± 7.2	81.5 ± 7.1	<0.001	5.816
Female	2,862 (60.6)	781 (54.9)	<0.001	14.381
CMI (Mean ± SD)	1.9 ± 1.7	2.3 ± 2.3	<0.001	-8.178
ICU stay	1,381 (29.0)	682 (47.7)	<0.001	173.625
Sepsis	563 (11.8)	223 (15.6)	<0.001	14.223
Palliative care	656 (13.8)	471 (32.9)	<0.001	271.855
Oral benzodiazepine	479 (10.1)	175 (12.2)	0.018	5.586
IV antipsychotics	387 (8.1)	382 (26.7)	<0.001	349.935
Oral antipsychotics	655 (13.7)	378 (26.4)	<0.001	127.592
IV opioids	1,642 (34.5)	701 (49.0)	<0.001	99.391
Oral opioids	1,003 (21.0)	273 (19.1)	0.110	2.557
Foley catheter used	1,225 (25.7)	494 (34.6)	<0.001	42.962
Alcohol related diagnosis	85 (1.8)	80 (5.6)	<0.001	61.655
Mortality	152 (3.2)	242 (16.9)	<0.001	348.529

Among patients without cognitive impairment admitted during the same three-year period [n=14,344 (69.8%)], only 1,468 (10.2%) received IV lorazepam (Table 2). The group who received IV lorazepam was not different in mean age but had a slightly higher percentage of women. The IV lorazepam group had a higher CMI, more often had an ICU stay, sepsis, and used palliative care services. The percentages of patients who received oral BDZs, IV antipsychotics, oral antipsychotics, and IV opioids were also higher in this group. Foley catheter use was slightly higher in the IV lorazepam group. Finally, the mortality rate in the IV lorazepam group was higher [N=470; (32%)] compared to those who did not receive IV lorazepam [N=265; (2.1%)] (P<0.001) (Table 2), a roughly fifteen-fold difference. 

**Table 2:** Descriptive characteristics of patients without cognitive impairment admitted to a community-based hospital, 2017 through 2019 (n = 14,344). The data in columns 2 and 3 have been represented as N (%) except for age and CMI, for which the data have been represented as Mean±SD. A two-sample t-test was used to test for differences in the mean of continuous variables and a chi-square test was used to test for differences in the frequency distributions of the categorical variables (column 5). P-values are shown in column 4 and considered significant if p<.05. Abbreviations: IV: intravenous; SD: standard deviation; CMI: case mix index; ICU: intensive care unit.

Characteristics	No IV Lorazepam (n = 12,876)	IV Lorazepam (n = 1,468)	P-value	T-test and chi-test values
	n (%)	n (%)		
Age (y) (Mean ± SD)	78.8 ± 6.6	78.7 ± 6.7	0.475	0.714
Female	6,992 (55.0)	859 (58.9)	0.004	8.087
CMI (Mean ± SD)	1.8 ± 1.5	2.1 ± 2.6	<0.001	-6.846
ICU stay	3,155 (24.5)	764 (52.0)	<0.001	503.355
Sepsis	682 (5.3)	129 (8.8)	<0.001	30.103
Palliative care	563 (4.4)	343 (23.4)	<0.001	803.314
Oral benzodiazepine	1,116 (8.7)	201 (13.7)	<0.001	39.901
IV antipsychotics	99 (0.7)	73 (5.0)	<0.001	196.571
Oral antipsychotics	251 (2.0)	67 (4.6)	<0.001	41.558
IV opioids	5,773 (44.8)	870 (59.3)	<0.001	110.341
Oral opioids	4,106 (31.9)	311 (21.2)	<0.001	70.841
Foley catheter used	2,596 (20.2)	338 (23.0)	<0.010	6.639
Alcohol related diagnosis	0	0	-	-
Morality	265 (2.1)	470 (32.0)	<0.001	2,400

Table 3 details the unadjusted and adjusted results of the logistic regression used to model the association between IV lorazepam use and mortality among patients with and without cognitive impairment. Among patients with cognitive impairment, those who received IV lorazepam had an increased aOR of mortality compared to those who did not receive IV lorazepam (aOR: 3.37; 95% CI: 2.52-4.50) after controlling for other covariables. Among patients without cognitive impairment, those who received IV lorazepam also had an increased aOR of mortality (aOR: 7.72; 95% CI: 6.09-9.79) after controlling for other covariables. 

**Table 3:** Results of the logistic regression analysis to evaluate the association between IV lorazepam use and mortality among patients admitted to a community-based hospital with and without cognitive impairment, 2017 through 2019. The unadjusted association between IV lorazepam use and mortality was estimated by fitting a logistic regression model where the primary exposure of interest was IV lorazepam use and the primary outcome of interest was mortality. The adjusted association between IV lorazepam use and mortality was calculated by including additional variables in the logistic regression model that were selected to statistically control for other factors that may influence the magnitude of the unadjusted estimate of association. Covariables were selected by first fitting an adjusted model that included all significant variables from the univariate analysis (i.e., the full model), then fitting a series of reduced models -- each omitting one of the candidate variables -- and then evaluating the independent contribution of the omitted variable to the full model using a likelihood ratio test. Note: Although some of the unadjusted variables had significant ORs, after logistic regression analyses, they did not make the final multivariable model. *Statistically significant variables as 95% CI does not include 1. Abbreviations: IV: intravenous; CMI: case mix index; ICU: intensive care unit; OR: odds ratio; CI: confidence interval; NI: “not included” in the final multivariable model.

Variables	Patients with cognitive impairment (n = 6,197)		Patients without cognitive impairment (n = 14,344)	
	Unadjusted OR (95% CI)	Adjusted OR (95% CI)	Unadjusted OR (95% CI)	Adjusted OR (95% CI)
IV Lorazepam	6.18 (5.00, 7.65)*	3.37 (2.52, 4.50)*	22.41 (19.03, 26.40)*	7.72 (6.09, 9.79)*
Age (years)	1.00 (0.99, 1.02)	NI	1.05 (1.04, 1.06)*	1.05 (1.03, 1.06)*
Male	1.38 (1.12, 1.69)*	NI	1.14 (0.99, 1.33)	NI
CMI	1.17 (1.13, 1.21)*	NI	1.05 (1.01, 1.09)*	0.90 (0.86, 0.95)*
ICU stay	6.52 (5.17, 8.22)*	3.09 (2.38, 4.01)*	6.34 (5.40, 7.44)*	3.35 (2.76, 4.08)*
Sepsis	4.29 (3.43, 5.36)*	2.39 (1.83, 3.13)*	3.01 (2.41, 3.75)*	2.27 (1.69, 3.04)*
Palliative care	10.77 (8.65, 13.42)*	4.72 (3.69, 6.04)*	13.95 (11.79, 16.51)*	5.35 (4.28, 6.68)*
Oral benzodiazepine	0.39 (0.24, 0.63)*	0.50 (0.30, 0.85)*	0.36 (0.25, 0.54)*	0.40 (0.26, 0.63)*
IV antipsychotics	0.74 (0.53, 1.04)	NI	1.65 (0.95, 2.87)	NI
Oral antipsychotics	0.31 (0.20, 0.47)*	0.28 (0.17, 0.44)*	0.79 (0.45, 1.38)	NI
IV opioids	3.87 (3.11, 4.82)*	1.90 (1.42, 2.54)*	2.18 (1.86, 2.54)*	NI
Oral opioids	0.56 (0.41, 0.75)*	0.56 (0.39, 0.80)*	0.21 (0.17, 0.28)*	0.41 (0.31, 0.55)*
Foley catheter used	1.36 (1.05, 1.62)*	NI	0.85 (0.70, 1.03)	NI
Alcohol related diagnosis	0.75 (0.36, 1.53)	NI	-	-

Among patients with cognitive impairment, other variables with increased aORs included ICU stay (aOR 3.09; 95% CI: 2.38-4.01), sepsis (aOR 2.39; 95% CI: 1.83-3.13), and palliative care (aOR 4.72; 95% CI: 3.69-6.04). Among patients without cognitive impairment, increased aORs were also seen for ICU stay (aOR 3.35; 95% CI: 2.76-4.08), sepsis (aOR 2.27; 95% CI: 1.69-3.04), and palliative care (aOR 5.35; 95% CI: 4.28-6.68) (Table 3).

For medications other than IV lorazepam, only the use of IV opioids among patients with cognitive impairment was associated with an increase in aOR (Table 3).

Although the logistic regression analyses controlled for covariables, since there is potential for significant overlap of use of IV lorazepam among patients in the ICU and those who received palliative care, all analyses were repeated excluding these patients. As seen in Table 4, the aOR of mortality among patients with cognitive impairment was highest among patients with sepsis (10.61; 95% CI: 5.66-19.88) but aOR for IV lorazepam remained high (4.09; 95% CI: 2.17-7.69). For those without cognitive impairment, aOR for IV lorazepam was higher than all other variables, 18.82 (95% CI: 13.39-26.46).

**Table 4:** Results of the logistic regression analysis to evaluate the association between IV lorazepam and mortality among older patients, both with and without cognitive impairment, admitted to a community-based hospital from 2017 through 2019 (excluding ICU and palliative care patients) (n = 13,671). The unadjusted association between IV lorazepam use and mortality was estimated by fitting a logistic regression model where the primary exposure of interest was IV lorazepam use and the primary outcome of interest was mortality. The adjusted association between IV lorazepam use and mortality was calculated by including additional variables in the logistic regression model that were selected to statistically control for other factors that may influence the magnitude of the unadjusted estimate of association. Covariables were selected by first fitting an adjusted model that included all significant variables from the univariate analysis (i.e., the full model), then fitting a series of reduced models -- each omitting one of the candidate variables -- and then evaluating the independent contribution of the omitted variable to the full model using a likelihood ratio test. Note: Although some of the unadjusted variables had significant ORs, after logistic regression analyses, they did not make the final multivariable model. *Statistically significant variables as 95% CI does not include 1. Abbreviations: IV: intravenous; CMI: case mix index; ICU: intensive care unit; OR: odds ratio; CI: confidence interval; NI: “not included” in the final multivariable model.

Variables	Patients with cognitive impairment (n = 3,633)		Patients without cognitive impairment (n = 10,038)	
	Unadjusted OR (95% CI)	Adjusted OR (95% CI)	Unadjusted OR (95% CI)	Adjusted OR (95% CI)
IV Lorazepam	3.13 (1.71, 5.73)*	4.09 (2.17, 7.69)*	18.83 (13.81, 25.67)*	18.82 (13.39, 26.46)*
Age (years)	1.10 (1.05, 1.14)*	1.11 (1.06, 1.16)*	1.09 (1.07, 1.11)*	1.08 (1.06, 1.11)*
Male	0.88 (0.48, 1.62)	NI	1.19 (0.88, 1.60)	NI
CMI	1.17 (0.92, 1.50)	NI	0.05 (0.03, 0.10)*	0.13 (0.08, 0.24)*
Sepsis	8.89 (4.85, 16.29)*	10.61 (5.66, 19.88)*	1.18 (0.57, 2.41)*	4.05 (1.81, 9.06)*
Alcohol related diagnosis	-	-	-	-
Foley catheter used	1.11 (0.56, 2.20)	NI	0.37 (0.20, 0.67)*	NI
Oral benzodiazepine	0.18 (0.24, 1.28)	NI	0.06 (0.01, 0.41)*	0.05 (0.01, 0.34)*
IV antipsychotics	0.93 (0.33, 2.60)	NI	1.16 (0.16, 8.43)	NI
Oral antipsychotics	0.49 (0.18, 1.39)	NI	-	NI
IV opioids	0.89 (0.46, 1.73)	NI	1.29 (0.95, 1.74)	NI
Oral opioids	0.38 (0.14, 1.06)	NI	0.12 (0.06, 0.24)*	0.29 (0.14, 0.57)*

## Discussion

In this large exploratory retrospective study of older hospitalized patients, we found that use of IV lorazepam was high among those with cognitive impairment, approximately 23% (N=1430/6197), and was associated with a three-fold increased risk of mortality after adjusting for other covariables. The study also found that although the use of IV lorazepam was lower among patients without cognitive impairment, approximately 10% (N=1468/14,344), it was associated with a seven-fold increased risk of mortality after adjusting for other covariables.

There are several possible reasons for these associations. IV lorazepam may cause or worsen delirium, and delirium and delirium severity are associated with increased mortality risk [6,12]. The use of IV lorazepam might also be a marker of agitation (for which lorazepam is still widely used) [15], and agitation may impede workup and treatment of acute medical illnesses, which may lead to negative outcomes such as mortality [16].

Another reason for the use of IV lorazepam in the hospital setting is related to procedures such as MRI. Up to 15% of patients have difficulty completing imaging procedures due to claustrophobia or restlessness and may receive pre-procedural medication. IV lorazepam is the most commonly used sedative in this situation and older patients are at higher risk for oversedation and medication dose-stacking, leading to serious negative outcomes [17].

Although most providers are aware that one of the main side effects of IV Lorazepam is sedation, many are unaware that the half-life of IV lorazepam is 12-15 hours and ~85% is protein-bound [18]. So even one dose in frail older patients, with or without cognitive impairment, might start a cascade of other events such as lethargy, immobility, and decreased oral intake leading to poor outcomes, including increased risk of pneumonia and mortality [17-19]. However, as discussed below, our database did not allow us to determine the dosage or duration of IV lorazepam. 

Another less common side effect of IV lorazepam is a paradoxical reaction, characterized by “irritability” and “excitability” which may lead to using physical or chemical restraints starting a cascade of decline as described above [18].

One of the surprising findings of this study is that the aOR for mortality was much higher among older patients without cognitive impairment compared to those with cognitive impairment. One might expect that those with cognitive impairment would be more susceptible to the central nervous system actions of lorazepam, and thus more likely to experience serious side effects. However, this was not the case. One possible explanation for this contradictory finding is that some patients categorized as “without cognitive impairment” may have had cognitive impairment, whether dementia or delirium, that was undiagnosed, not documented, or not identified by the hospital coders. But is it also possible that there might be something inherently different in lorazepam compared to other BDZs? A translational cancer mechanism research study examined the association between cancer-free survival and two of the most commonly used BDZs, lorazepam and alprazolam, among patients in their cancer center database. The study found that lorazepam, but not alprazolam, was associated with increased mortality for most types of cancer. The researchers then utilized mouse models to study changes in the tumor environment. They found that lorazepam, not alprazolam, stimulated inflammatory signaling (IL6 production) and ischemic necrosis [20].

On review of the literature, it was difficult to find similar studies to ours. We found two small studies that focused on the use of BDZs and hospitalized older patients. In one study of 212 older patients hospitalized with delirium, the use of lorazepam was not associated with an increased risk of mortality [7] while another study of 133 older patients admitted with delirium found a higher mortality rate among BDZ users [9]. These studies did not provide the details needed to determine if the lorazepam or BDZ was IV or oral.

In the ICU setting, IV lorazepam, an independent risk factor for daily transition to delirium [6], is associated with increased mortality compared to other sedatives and is not recommended for sedation or agitation [12].

An observational study on 30-day mortality risk associated with the use of pre-operative IV BDZ use did not find an increased risk of mortality. This study did not detail which IV BDZs were used, but midazolam is most often used in this setting because of its short half-life [8].

Park and colleagues analyzed four IV anesthetics (propofol, midazolam, diazepam, lorazepam) from a national database in South Korea. They found lorazepam had the highest overall proportion of adverse drug reactions (ADRs), the highest proportion of serious ADRs, and was second highest (after propofol) related to deaths [21].

Larger studies about the risks of BDZs are community-based and evaluate oral BDZ use. In a nationwide cohort of 54,958 adults aged>65 years, BZD use was associated with all-cause mortality in short-term and chronic users [11]. Another large cohort study of older adults without dementia did not find an association between BDZ intake and risk of all-cause mortality over a 12-year period after adjustment for psychiatric disorder [10]. In the current study, IV lorazepam was associated with mortality even after adjusting for IV and oral antipsychotic use.

There are some strengths to this study. To our knowledge, there are no previous studies that have examined the use of IV lorazepam among such a large hospital cohort of consecutively admitted older patients. Furthermore, most previous studies have not differentiated between patients with and without cognitive impairment.

There are certain limitations to the current study. The primary limitation was the lack of contextual information about IV lorazepam in our data. For example, our data did not include the reason for IV lorazepam use. Based on our clinical experience [3], the most common reasons for IV lorazepam use are agitation, restlessness, and “on-call for MRI.” Two other uses in the hospital include palliative care (which we have controlled for as noted above) and alcohol withdrawal syndrome. Alcohol-related diagnosis was a small percentage of patients in the current study. 

Additionally, our data did not include dosage, the number of doses, duration, or the total amount of IV lorazepam used. Thus it was not possible to determine whether the associations were dose-dependent.

Another important limitation of the data used in this study was the absence of causes of death from the electronic medical record database. Also, our findings are based on a database from a single hospital.

This type of retrospective observational study is subject to a bias called confounding by indication. For our study, the bias has to do with the possibility that the indication to use IV lorazepam, not the IV lorazepam itself, is the risk factor associated with mortality. For example, the overlap between palliative care, ICU stay, and the indications to use of IV lorazepam potentially complicates the interpretation of our results. In a sensitivity analysis that excluded patients with palliative care and an ICU stay, the association between IV lorazepam and mortality persisted, but there still exists some bias in this analysis.

Concerning palliative care, one study of patients with advanced cancer in an acute palliative care ward with delirium and agitation found that patients receiving IV lorazepam (3 mg) plus IV haloperidol had lower RASS score when compared to placebo plus haloperidol, but survival did not differ between the two groups. These patients were younger than our population (mean age 62) and patients were excluded if they had dementia [13].

In another study of inpatient hospice and palliative care patients, the use of BDZs (unclear if oral or IV) was associated with an increased risk of death if terminal delirium was present, but not in patients without terminal delirium [14].

## Conclusions

Our exploratory study highlights the common use of IV lorazepam in hospitalized older adults and the possible increased risk of mortality associated with its use in both cognitively impaired and cognitively intact older adults. Given the important limitations of our data, especially the lack of data on the dosage and duration of IV lorazepam, these results should be interpreted with caution. Also, importantly, the findings here are associations, not causal. Nevertheless, these results warrant additional studies designed to rigorously investigate this topic. If the current findings are replicated, they have the potential to improve best practices and potentially prevent premature death.

## Figures and Tables

**Figure 1:**
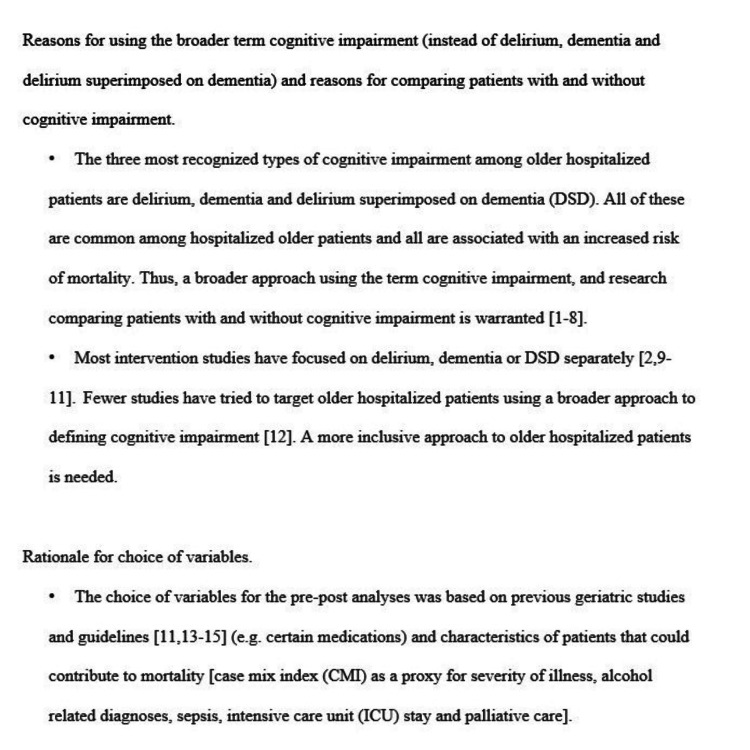
Reasons for using the broader term cognitive impairment and reasons for comparing patients with and without cognitive impairment.

## Appendices

Appendix one

**Table 5:** International Classification of Diseases (ICD-10) Diagnostic Codes Associated with Cognitive Impairment

Diagnostic Code	Diagnosis Description
F0150	Vascular dementia without behavioral disturbances
F0280	Dementia in other diseases classified elsewhere without behavioral disturbances
F0281	Dementia in other diseases classified elsewhere with behavioral disturbances
F0390	Unspecified dementia without behavioral disturbances
F0391	Unspecified dementia with behavioral disturbances
F05	Delirium due to known physiological condition
F062	Psychotic disorder with delusions due to known physiological condition
F064	Anxiety disorder due to known physiological condition
F068	Other mental disorders due to known physiological condition
F10121	Alcohol abuse with intoxication delirium
F10221	Alcohol dependence with intoxication delirium
F10231	Alcohol dependence with withdrawal delirium
F10921	Alcohol use, unspecified with intoxication delirium
F1123	Opioid dependence with withdrawal
F11921	Opioid use, unspecified with intoxication delirium
F13239	Sedative/hypnotic/anxiolytic dependence with withdrawal, unspecified
F19121	Other psychoactive substance abuse with intoxication delirium
F19921	Other psychoactive substance use, unspecified with intoxication delirium
F19931	Other psychoactive substance use, unspecified with withdrawal delirium
G300	Alzheimer’s disease with early onset
G301	Alzheimer’s disease with late onset
G309	Alzheimer’s disease, unspecified
G4751	Confusional arousals
G92	Toxic encephalopathy
G9340	Encephalopathy, unspecified
G9341	Metabolic encephalopathy
G9349	Other encephalopathy
P9161	Mild hypoxic ischemic encephalopathy [HIE]
R402240	Coma scale, best verbal, confused conversation, unspecified time
R410	Disorientation, unspecified
R4182	Altered mental status, unspecified
R451	Restless and agitation
T8189XA	Other complications of procedures, NEC, init
T8189XD	Other complications of procedures, NEC, subs
T8189XS	Other complications of procedures, NEC, sequela
Z87898	Personal history of other specified conditions

Appendix two
